# From Neural Crest to Definitive Roof Plate: The Dynamic Behavior of the Dorsal Neural Tube

**DOI:** 10.3390/ijms22083911

**Published:** 2021-04-10

**Authors:** Dina Rekler, Chaya Kalcheim

**Affiliations:** Department of Medical Neurobiology, Institute of Medical Research Israel-Canada (IMRIC) and the Edmond and Lily Safra Center for Brain Sciences (ELSC), Hebrew University of Jerusalem-Hadassah Medical School, P.O.Box 12272, Jerusalem 9112102, Israel; dinarekler@gmail.com

**Keywords:** BMP, cell cycle, dorsal interneurons, epithelial to mesenchymal transition, neural crest, neural tube, definitive roof plate, somite, Wnt

## Abstract

Research on the development of the dorsal neural tube is particularly challenging. In this highly dynamic domain, a temporal transition occurs between early neural crest progenitors that undergo an epithelial-to-mesenchymal transition and exit the neural primordium, and the subsequent roof plate, a resident epithelial group of cells that constitutes the dorsal midline of the central nervous system. Among other functions, the roof plate behaves as an organizing center for the generation of dorsal interneurons. Despite extensive knowledge of the formation, emigration and migration of neural crest progenitors, little is known about the mechanisms leading to the end of neural crest production and the transition into a roof plate stage. Are these two mutually dependent or autonomously regulated processes? Is the generation of roof plate and dorsal interneurons induced by neural tube-derived factors throughout both crest and roof plate stages, respectively, or are there differences in signaling properties and responsiveness as a function of time? In this review, we discuss distinctive characteristics of each population and possible mechanisms leading to the shift between the above cell types.

## 1. Introduction

Pattern formation during embryonic development relies on precursor cells adopting one of several alternative fates. These decisions are determined by a combination of extrinsic signals, such as morphogen gradients, intercellular interactions mediated by Notch-Delta activities, and cell-intrinsic factors that respond to the precedent signals and comprise a downstream transcriptional regulatory network. Together, these are believed to specify particular cell identities [1,2,3].

The generation of distinct cell identities along the dorsoventral axis of the neural tube (NT) is an excellent model for investigating cell decisions in time and space. It involves the integration of opposing concentration gradients of Sonic hedgehog ventrally and of Bone Morphogenetic Protein (BMP) and Wnt dorsally [4,5,6,7]. In the dorsal NT, BMPs and Wnts are first secreted by the non-neural ectoderm [8,9] and later are produced in the dorsal NT itself to establish a signaling gradient that controls the sequential specification of neural crest (NC), roof plate (RP) and dorsal-most interneuron progenitors [10,11,12].

Hence, a major challenge is that the above transitions are intrinsically dynamic, an outcome of time and stimulation. At first, the dorsal domain of the NT is transiently populated by premigratory NC cells. These exit the NT to form a rich collection of cell types, such as sensory and autonomic neurons, satellite cells and Schwann cells of the peripheral nervous system as well as pigment cells, ectomesenchyme and endocrine derivatives whose combination varies along the neuraxis [13,14]. Subsequently, the dorsal NT midline is replaced by the definitive RP of the spinal cord, which becomes flanked ventrally by dorsal interneuron populations [12,14,15,16].

The sequential generation of the above cell types raises fundamental questions on the dynamics of dorsal NT behavior. When does fate segregation occur; prior to cell emigration due to cell-cell interactions or to a graded response to morphogens, and/or during migration when multipotent NC cells may be instructed by environmental cues to form specific derivatives? Evidence points to a heterogeneous nature of the NC at the population level, being a mixture of both fate restricted and multipotent progenitors at the various stages. As such, the measured proportion of cells with different degrees of commitment is likely to vary as a function of the methods used to follow cell lineages, on animal species, and on specific axial levels considered at progressive stages. Along this line, analysis at a given axial level over time showed a stereotypic pattern of migration of NC progenitors followed by ordered target colonization [17,18]. In a few studies, this ordered cellular behavior was consistent with the existence of fate-restricted precursors already in the premigratory domain [14,17,19,20,21]. Mechanistically, the question arises whether fate restriction is accounted for by a direct differentiation of multipotent progenitors into definitive cell types or by a progressive mechanism involving cells with intermediate specification states. This is still an ongoing debate, and is only briefly outlined here; the reader is referred to additional relevant literature [15,22,23,24].

Despite this still active controversy, significant progress in other aspects of NC ontogeny, such a cell emigration, migration and differentiation, has been achieved over the years [13,25,26,27,28]. Virtually nothing is known, however, about how the production and emigration of NC cells reach an end. Furthermore, are the stop signals for NC production sufficient for specifying the succeeding RP or are there in addition specific RP inducers? When is the definitive RP specified? Where in the neuroepithelium do RP progenitors originate? How is the NC to RP transition regulated along the neuraxis in different species? Understanding the cellular and molecular logic of the transition between peripheral (NC) and central (RP) branches of the nervous system is an extremely exciting, yet mostly undiscovered topic.

## 2. Neural Crest-Premigratory Behavior and Cell Emigration

The dorsal region of the NT that contains premigratory NC cells is a pseudostratified epithelium in which progenitor cells undergo interkinetic nuclear migration and exhibit typical patterns of cell proliferation [29,30]. This epithelial state is transient, as progenitor cells either delaminate progressively or fully dissociate to generate migratory mesenchymal cells. In the dorsal NT of avians at trunk levels, EMT and cell delamination are gradual events lasting about two consecutive days, during which the dorsal NT downregulates N-cadherin protein expression, yet preserves its general epithelial structure [31]. In contrast, cranial NC cells exit the neural folds or the closed NT as a cohesive group of progenitors which undergo only a partial EMT and rapidly split into distinct streams of cells [32,33]. Accumulating evidence suggests that the molecular networks controlling EMT at each level are different (reviewed in [32,34]. In this section, we ask what is known about the behavior of premigratory NC progenitors in terms of cellular traits, possible molecular heterogeneity and fate restriction. Furthermore, we briefly elaborate on the multileveled nature of regulation of NC EMT.

### 2.1. Neural Crest Progenitors Residing in the Dorsal NT

**Figure 1:**
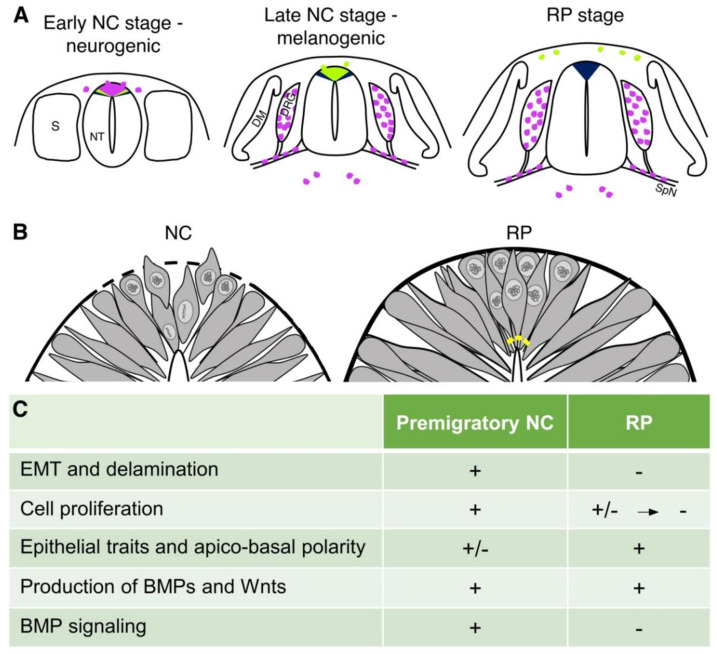
The dynamics of the dorsal NT—from premigratory NC to definitive RP. (**A**) Schematic representation of three consecutive stages. The early emigrating NC (purple) is neurogenic—premigratory NC cells at trunk levels express *Foxd3*, *Snai2*, *Sox9*, etc; upon delamination they migrate dorso-ventrally to give rise mainly to neural and glial derivatives. A subset of NC-derived Schwann cell progenitors also generates hypaxial melanocytes. The late NC (green) is melanogenic—premigratory NC cells downregulate expression of the above genes, and as they leave the NT they migrate dorso-laterally and differentiate into epaxial melanocytes. RP progenitors (blue) are generated ventral to the premigratory NC and adopt their final position following the end of NC emigration. (**B**) Differences in cellular behavior between premigratory NC cells and definitive RP cells. Shortly prior to emigration, NC cells loose epithelial traits and their nuclei are distributed throughout the apico-basal extent of the dorsal NT (left panel). Upon formation of the RP, cells regain epithelial traits and apico-basal polarity with nuclei mainly concentrated at the basal half of the epithelium. Yellow squares represent apical adherens junctions (right panel). (**C**) A summary of various characteristics of premigratory NC and RP stages (see text for details). Abbreviations; DM, dermomyotome, DRG, dorsal root ganglion, NC, neural crest; NT, neural tube; S, somite; SpN, spinal nerve.

Discrete labeling of the dorsal NT in several species revealed that, following EMT, NC cells migrate in a stereotypic manner and colonize their peripheral targets in a general ventral to dorsal sequence [17,18,35,36,37,38]. In mouse and Xenopus embryos, both ventral and subectodermal pathways are invaded simultaneously [39,40]. Such a stereotypic migration of NC cells in the periphery, raised the question of the mechanisms that operate in the premigratory domain of the NT. One possibility is that the premigratory domain acts as a reservoir of proliferating stem cells that, upon cell division, generate one emigrating cell and another daughter cell that remains in the tube. Alternatively, proliferating premigratory progenitors could leave the NT in an ordered and sequential fashion via a non-stem cell mechanism. To address these alternative models, small cell populations in the avian dorsal tube at flank regions of the axis were lineage traced. Most labeled cells delaminated without leaving residual progeny in the neuroepithelium excluding the asymmetric mode of cell emigration ([17] and see also [41]). Furthermore, upon initiation and progression of cell exit, a corresponding ventral to dorsal relocation of premigratory neuroepithelial progenitors was monitored until exhaustion of the prospective NC pool [17] (Figure 1A). This ventro-dorsal cellular progression followed by cell delamination was later confirmed using a photoconvertible fluorescent protein [42]. Together, this sequence of events suggests that initial NC delamination generates the force driving relocation of epithelial progenitors towards the dorsal midline region. Consequently, a progressive narrowing of the pre-migratory NC domain occurs until its replacement by the definitive RP. Thus, the dorsal midline area of the NT is a “dynamic” epithelium and acts as a transition zone for the gradual inflow and departure of cells [15,17,19].

The dynamic yet precise cellular behavior described above would indicate that the dorsal NT is molecularly heterogeneous, either at the spatial and/or temporal levels. Several lines of evidence lend support to this notion. Early premigratory NC progenitors have been shown to express *FoxD3*, *Sox9* and *Snai2,* whereas late progenitors do not [19]. Furthermore, analysis of progenitors expressing a specific *Foxd3* reporter, confirmed that neural progenitors are *Foxd3*-positive but prospective melanoblasts downregulate *Foxd3* and segregate from neural lineages already before emigration. Moreover, when the normal downregulation of *Foxd3* is prevented by gene misexpression at a late stage corresponding to the end of neural lineage production, the late-emigrating precursors failed to upregulate the melanogenic markers *Mitf* and MC-1 and the guidance receptor *Ednrb2*, generating instead glial cells that expressed P0 and *Fabp*. In a reciprocal experiment, loss of Foxd3 function in mouse NC resulted in ectopic melanogenesis in the dorsal tube, in sensory ganglia and along ventral roots [19,43]. These results suggest that a timely downregulation of FoxD3 gene activity in the dorsal NT is necessary for the switch between neural and melanocytic phases of NC development. In this regard, a previous study also highlighted the need for downregulating Foxd3 to enable upregulation of *Mitf* and melanogenesis, albeit in this study *Foxd3* was misexpressed at very early stages, thus comprising both neural as well as melanogenic precursors, rather than attaining exclusively the prospective pigment cell subset [44]. Since, similar to the downregulation of *Foxd3*, *Snai2* and *Sox9* are also lost from the dorsal NT prior to melanoblast emigration, it is likely that the latter two, along with *Foxd3*, form part of a network that influences neural vs. melanocyte development. The differential expression of these three genes to prospective neural lineages but not to melanoblasts, highlights a temporal sequence of molecular differences between the above fates apparent already at the premigratory stage.

Additional evidence for molecular heterogeneity in the dorsal NT stems from an unbiased hierarchical clustering of 35 genes that was performed at a cranial level of the avian axis. This analysis revealed five distinguishable clusters that mapped to different domains of the dorsal neural primordium. For example, a central portion expressed a combination of NC, pluripotency and differentiation markers of NC, whereas a more lateral subdomain exhibited “NC only” genes, together highlighting spatial differences within the premigratory region of the NT [45]. In addition, by characterizing transcriptional signatures and cis-regulatory elements at both global and single cell levels, the presence of segregated subpopulations was already apparent at the premigratory stage in the head of avian embryos [46]. A recent study performed in mice further emphasized that premigratory cranial NC cells are molecularly heterogeneous and carry positional information that reflects their origin in the neuroepithelium. Most notably, it appears that this information is transiently erased during emigration, as delaminating cells were found to be transcriptionally uniform. The authors proposed that this would allow ectoderm-derived NC cells to generate mesenchymal derivatives, unique to the head region. Consistently, the latter are produced from a subset of cells that re-express the pluripotency factor *Oct4*, which acts in this context, on specification and survival of the ectomesenchyme. Along this line, no re-expression of *Oct4* was detected along more caudal regions of the axis corresponding to trunk levels, suggesting a different mechanistic scenario [47].

Similarly, using an *EdnrB* enhancer, a comprehensive temporal map of the chromatin and transcriptional landscape of vagal-level NC cells revealed the existence of three clusters: neural, neurogenic and mesenchymal, each predetermined epigenetically prior to NC delamination [21]. Recently, single cell RNA sequencing in zebrafish embryos revealed premigratory subpopulations already expressing genes associated with multiple differentiated melanocytic fates [48]. In mice, early genes encoding for competing cell programs coactivate in single progenitors from a premigratory stage onward; this would represent the first phase preceding fate bias, apparent during delamination, and subsequent commitment [49]. Availability of a new resource of premigratory NC-specific genes in quail embryos [50] will enable in the near future to examine in more detail the evolution of molecular heterogeneity within the dorsal neuroepithelium at trunk levels of the axis both prior to- and during progressive cell exit.

Is there any relationship between the observed molecular heterogeneity of premigratory NC progenitors described above, the precise localization of a cell in the dorsal NT and the acquisition of distinct fates by the migrating cells? One extreme possibility is that there is no relationship between the localization of presumptive progenitors in the dorsal tube, the sequence of their emigration and their final fates. McKinney et al. suggested this possibility for most trunk derivatives except for sympathetic progenitors [42]. A second possibility is that spatial and/or temporal information in the dorsal NT itself biases an initial segregation of NC cells into some of their derivatives. The latter was suggested for the development of neural vs. epaxial melanocyte precursors, a choice apparently independent of the migratory routes followed by the cells [19]. In addition, single cell labeling of the early dorsal NT midline corresponding to the production of autonomic progenitors, the first to exit the NT [17], revealed that clones contained both sympathetic neurons and chromaffin fates, suggesting that sympathoadrenal cells share a common progenitor in the premigratory zone. However, the clones detected contained no additional cell types characteristic of trunk NC [51]. These and additional results suggest that, at least for derivatives of the thoracic NC, a link exists between initial cell localization in the dorsal NT, time of emigration and final localization/fate. The reader is referred to previous reviews thoroughly discussing this important and still debated issue [14,15].

### 2.2. Exiting the Neural Tube to Engage in Cell Migration

One of the hallmarks of NC development is the ability of premigratory precursors to exit the neuroepithelium via a process of epithelial-to-mesenchymal transition (EMT) followed by cell delamination and generation of cellular motility [13]. Notably, EMT and cell delamination are closely associated with the cell cycle. In the trunk level of the axis, where cells exit the NT as individual progenitors over a relatively long period of time, it was shown that about 85% of them synchronize to the S-phase of the cell cycle during emigration and undergo actual cell division once they left the NT. Furthermore, the transition from G1 to S was demonstrated to be crucial for NC emigration, suggesting that during this stage, cells make key decisions, such as to undergo EMT, based on complex signaling with their microenvironment [30]. As a result, of this synchronization to the S phase during delamination, the immediate premigratory domain exhibits a lower percentage of cells in the doubling phase of their DNA [30], an observation associated with expression of *Snail* genes in this domain [52]. In the NC, in gastrulation, in the invasive front of various carcinomas and in additional situations, this phenomenon was taken as evidence that profound morphological changes, such as those taking place during EMT, are somewhat incompatible with high cell proliferation ([52] and refs. therein).

In contrast, no synchronization to the S-phase seems to be required for emigration of cranial NC cells [53], perhaps because the latter undergo partial EMT during exit from the NT and only adopt full mesenchymal properties during advanced migration [54]. Yet, electroporation of a dominant-negative version of the p53 tumor suppressor increased cranial NC number and EMT/delamination. Investigating the underlying molecular mechanisms revealed that p53 coordinates cell cycle gene expression and proliferation with EMT/delamination [55], further stressing an association between the above processes. Another study addressed the function of cMyc, a multifunctional protein involved in cell proliferation and invasiveness. cMyc is expressed in the avian premigratory cranial NC concomitant with the onset of EMT; loss of cMyc function was reported to reduce the number of premigratory cells and the extent and duration of EMT. However, cMyc did not directly affect cell cycle properties; instead, the authors proposed that cMyc acts both by affecting NC survival and also in vitro self-renewal [56].

Substantial evidence supports the notion that the process of EMT invokes the concerted action of signaling proteins with a network of transcription factors, affecting downstream cytoskeletal and cell adhesion properties [26,57], as well as the cell cycle properties described above. Concomitantly, cells degrade the overlying basement membrane in order to invade the extracellular matrix, processes considered to be essential for the acquisition of cell motility ([58] and refs. therein). Being such an essential and multifaceted process, it is expected that the regulation of NC EMT is highly complex.

A balance between BMP and its inhibitor noggin, in association with the developing somites, was found to underlie the emigration of trunk-level NC [10,59]. BMP induces EMT of NC by triggering *Wnt1* transcription that in turn promotes G1/S transition, a necessary step for delamination of trunk NC [60]. N-cadherin and Rho/Rac GTPases are also part of the BMP-dependent network of genes with activity on NC emigration [31,61,62]. Subsequently, it was found that dynamic counter-gradients of FGF8 and retinoic acid in the paraxial mesoderm affect NC EMT partly through the modulation of specific aspects of BMP and Wnt signaling [63]. A role for BMP and its antagonists was further reported to regulate mammalian NC survival and emigration [64].

Another important player in this genetic network is Yes-associated-protein (YAP), an effector of the Hippo pathway, that controls various aspects of development including cell proliferation, migration, survival and differentiation [65,66]. *YAP* is expressed and is active in premigratory NC of avian embryos. Gain of YAP function stimulates NC EMT, and attenuation of YAP inhibits cell exit. This is associated with reduced G1/S transition and enhanced apoptosis. Using specific in vivo reporters, loss of YAP function in the dorsal NT was found to inhibit BMP and Wnt activities whereas gain of YAP function stimulates these pathways. Reciprocally, inhibition of BMP or Wnt signaling downregulates YAP activity. In addition, YAP-dependent stimulation of NC emigration was compromised upon inhibition of either BMP or Wnt activities. These data established for the first time a positive bidirectional crosstalk between these pathways and incorporated YAP signaling into a BMP/Wnt-dependent molecular network responsible for emigration of trunk-level NC [67]. YAP signaling was also shown to mediate EMT of cranial NC downstream of metabolic remodeling towards enhanced aerobic glycolysis, a shift occurring prior to cell delamination [68]. Open questions remain as to whether YAP also affects survival and/or proliferation of cranial NC; whether BMP/Wnt enhance glycolysis of cranial-level NC progenitors, and whether the metabolic status of trunk NC cells undergoing EMT also changes, given that EMT at cranial and trunk levels of the axis differ significantly.

Transcription factors, regulated by the above signaling proteins, are an essential component of the molecular network leading to NC EMT and delamination [26,33,69]. Although many such genes define the premigratory state of NC cells at different axial levels [28,50], relatively few were directly shown to be involved in NC delamination. Perhaps the most salient example is *Snai2*, one of the earliest described genes in developmental EMT and metastasis [70,71]. Notably, *Snai2* does not appear to affect EMT of trunk NC [72,73], neither do Snail genes promote this process in the mouse NC [74]. However, *Snai2* plays a pivotal role in regulating EMT of NC precursors in the head, where *Cad6B* acts as a direct target of *Snai2* repression [75]. Furthermore, the adaptor protein PHD12 was shown to directly interact with Sin3A/histone deacetylase, which in turn interacts with *Snai2*, forming a complex at the *Cad6b* promoter [34]. In Xenopus, *Snai2/Slug* was shown to cooperate with the Polycomb repressive complex 2 (PRC2) to regulate various aspects of NC development including specification and EMT/migration [76]. Recently, the chromatin remodeler *Hmga1* was found to act both on NC specification at the neural plate border, and at a later stage, on NC emigration via canonical Wnt signaling [77]. Along this line, the Wnt modulator Draxin has been suggested to affect cranial NC EMT by remodeling the basement membrane upstream of *Snai2* [78]. The precedent studies are few examples highlighting the interaction between signaling and transcription factors, epigenetic mechanisms, downstream adhesion and matrix integrity in regulating the onset of cranial NC motility.

Less is known about transcription factors with effects on NC EMT at thoracic levels, and few examples will be provided here. *c-Myb* appears to regulate both the formation as well as EMT of NC cells downstream of BMP [79]. Furthermore, the combination of *Sox9*, *Snai2*, and *Foxd3*, but not each factor separately, effectively induced ectopic EMT on the dorsoventral extent of electroporated NTs, along with other traits of NC cells [80], but loss of these genes had no effect on NC EMT [43,74,81]. Zeb1 and Zeb2 are zinc finger transcription factors involved in cancer metastasis [82]. *Sip1*/*Zeb2*-defective mouse embryos revealed persistent E-cadherin expression in NC precursors that accumulate in the NT, indicating defects in EMT [83]. Involvement of Sip1/Zeb2 in the regulation of chick cranial NC delamination was also demonstrated by using antisense morpholino oligonucleotides [84]. In avian trunk NC cells, expression of *Zeb1* and *Zeb2* were found to overlap. By interfering with their expression using shRNAs, the authors showed that both factors share an equivalent stimulatory function on NC EMT [85]. Future studies should address the roles of additional transcription factors and their crosstalk with signaling and epigenetic mechanisms in regulating NC EMT at axial levels caudal to the head region. Such studies should also impact our understanding of cell metastasis in NC-derived tumors in which expression of NC-specific signatures may be correlated with either differentiative or aggressive properties [86].

## 3. From NC to RP-Differential Properties and Axial Level Variability in RP Morphology

The dorsoventral organization of the vertebrate central nervous system (CNS) is coordinated by two groups of cells known as organizers, the RP and the floor plate (FP). As such, both cell subsets are composed of post-mitotic cells with the nascent FP exiting the cell cycle earlier than RP [87]; furthermore, both cell types express the transcription factor *HES* in a non-cyclic, persistent manner and do not undergo neurogenesis [88,89]. Thus, by suppressing proliferation and neuronal differentiation, a rather constant amount of signal may be produced and maintained.

The RP has classically been considered the dorsal domain of the vertebrate NT along the entire rostro-caudal axis, where it produces morphogens responsible for dorsal cell fates, including BMPs [90,91,92,93,94,95] and wingless/Wnt proteins [96,97]. However, morphogens like BMPs and Wnts and transcription factors like *MafB*, *Msx*, *Lmx1a*/*b*, etc., which are considered as RP markers, are produced in the dorsal NT from closure of the neuroepithelium onward, including the early NC period [12,15,98,99]. In addition, the RP was shown to be induced by BMP4 and BMP7 [7], but the induced cells included both NC and RP. Since the dorsal NT at both NC and RP stages differs significantly both in terms of cell fates and cellular behaviors, we propose to discriminate between a NC stage and a definitive RP stage ([12] and see below).

Where do RP cells originate in the neuroepithelium? Our results [17] suggest that progenitors of the definitive RP are initially located ventral to the prospective NC. Initially, these cells are molecularly indistinguishable from presumptive NC, since they also express *Foxd3*, as evidenced by lineage analysis with a *Foxd3* reporter [19], and are still responsive to BMP, as revealed by the use of a BRE-GFP reporter. Upon NC emigration, prospective RP cells relocate dorsally towards their definitive midline position, and during this time they become refractory to BMP, downregulate *BMPR1A (Alk3)*, and cease to express *Foxd3* and the direct BMP target genes *Id2/3* (Figure 1). This is in spite the fact that RP cells continue synthesizing various BMP family members [12]. The initiation of Hes/*Hairy1* expression is associated with these events; the latter is initially evident in a band of cells localized ventrally to the *Foxd3*–positive domain; upon loss of BMP responsiveness and *Foxd3* transcription, Hairy1 expression and activity are evident in the dorsalmost NT domain [12]. Therefore, the dorsal NT is a dynamic area from which progressive NC emigration takes place until replacement by the definitive RP; this leads to the separation between central and peripheral branches of the nervous system [15]. As mentioned above, we sustain that the use of the term RP as the structure emerging upon NT closure is inappropriate, and therefore implement the term RP only when NC delamination has ended, and the segregation between CNS and PNS lineages is evident.

A pivotal question that emanates from the preceding findings is how does the dorsal NT transit from a NC to a definitive RP state? To address this question, it was first necessary to define key cellular properties that distinguish between these populations. Premigratory NC progenitors are mitotically active cells, whereas RP progenitors progressively withdraw from the cell cycle. Next, NC cells lose epithelial traits and apico-basal polarity shortly prior to emigration, revealing an incomplete laminin-containing basal lamina, loss of N-cadherin protein but not mRNA, disorganized ZO-1-positive tight junctions and Arl2b-positive cilia. In contrast, transition into the RP stage involves the regeneration of intercellular contacts and apico-basal polarity, suggesting that the latter structure regains epithelial traits [12,31] (Figure 1B,C).

**Figure 2:**
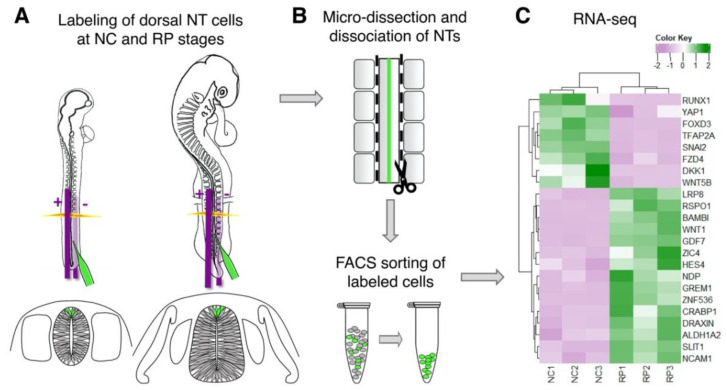
Schematic representation of a transcriptomic analysis comparing premigratory NC to RP. (**A**) Electroporation of GFP-DNA (green) was directed ventro-dorsally from the negative (-) to the positive (+) poles to label cells in the dorsal region of the quail NT at either the NC or the RP stage. (**B**) Transfected NTs were isolated, and dissociated into single cells, followed by FACS sorting. (**C**) RNA-seq analysis of fluorescent cells was performed in triplicate samples and reveals differential gene expression between premigratory NC and definitive RP cells.

Second, our understanding of RP formation is hindered by the lack of genes uniquely transcribed in either NC or RP populations. To overcome this limitation, a transcriptome analysis was performed at the trunk level of quail embryos comparing the dorsal NT at premigratory NC and at RP stages (Figure 2). In addition to many transcripts downregulated in RP when compared to NC, a selection of genes expressed in RP but not premigratory NC was uncovered. These included the RP-specific Spondin *Rspo1* [100] and *HES4*, the quail ortholog of chick *Hairy1*. In addition, the BMP member *Gdf7*, and the BMP antagonists *BAMBI* and *Gremlin*, the retinoic acid-associated genes *Raldh2* and *CRABP1*, and the chemorepellents of commissural axons *Draxin* and *Slit1*. Additional transcripts were evidenced that exhibited specific expression at the RP stage vis-à-vis the NC, yet a wider pattern that included additional NT regions. These genes included *Norrin (NDP)*, *LRP8*, *Znf536*, and *Zic4.* This recent RNAseq analysis provided a set of many spatiotemporal-specific genes appropriate for cell type identification and for functional studies [50]. Among the RP-specific genes, a subset was primarily expressed in the periphery of this structure and others in its center, highlighting a molecular heterogeneity within the RP at trunk levels of the axis whose biological significance remains to be investigated [50].

**Figure 3:**
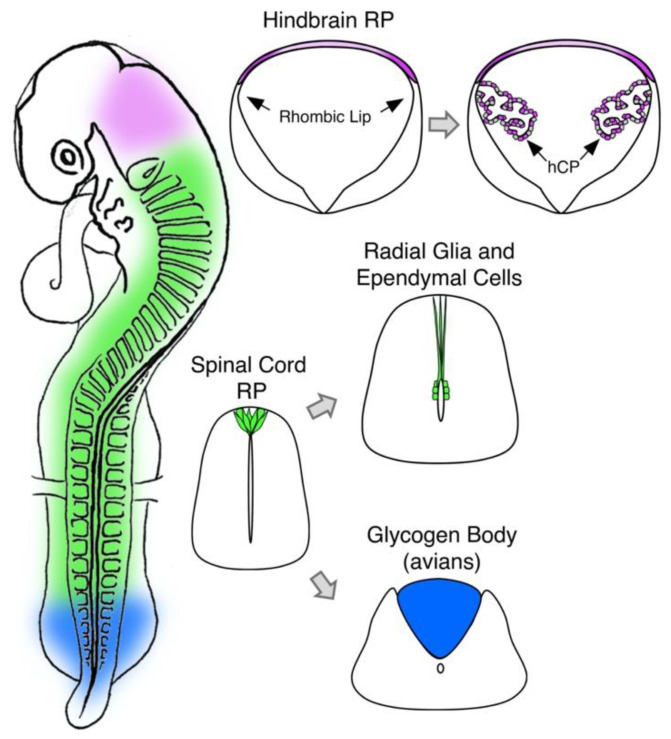
Differential properties of the RP at various axial levels. The hindbrain RP (purple) forms as an enlarged sheet of squamous epithelial cells bordered by the rhombic lip epithelium. The lateral domains of the hindbrain RP generate choroid plexus cells (hCP). The RP at spinal cord levels (green) is initially composed of pseudostratified epithelial cells, which are later transformed into radial glial cells that stretch and elongate as the lumen of the NT shrinks to become the central canal of the spinal cord. These radial glial cells were shown to give rise to a subset of ependymal cells lining the central canal. In birds, the RP at the lumbar level (blue) is transformed into an ovoid gelatinous glycogen body, which was proposed to serve as a physical barrier to dorsal midline crossing, thus enabling alternating gait as opposed to synchronous upper limb movement (see text for details).

A subject worth mentioning is the differing morphology of the RP along the neuraxis (Figure 3). Whereas along the dorsal midline of the spinal cord the RP is a relatively thin, wedge-shaped strip of cells, in the hindbrain, it is composed of an expanded sheet of cells, comprising three spatio-temporal fields differing in organization, proliferative state, and molecular traits. It was suggested that only two of the above fields contribute to the generation of the epithelial component of the choroid plexus [101,102]. Furthermore, the hindbrain RP is segmented along the rostro-caudal axis deriving from different rhombomeres with no intermixing [103]. Whether longitudinal cell mixing occurs at spinal cord levels of the RP remains unknown. This is particularly intriguing as their NC predecesors were shown to migrate longitudinally for a length of about two segments along the NT prior to initiating a dorso-ventral movement [104]. On the other hand, while we are beginning to understand the differential traits expressed in premigratory NC and RP at spinal cord levels, the equivalent knowledge for the hindbrain and other cranial regions that produce NC cells is still lacking.

In the hindbrain of chick embryos, a *Gdf7*-positive RP boundary was evidenced between the rhombic lip neuroepithelium and the RP proper. This boundary was shown to signal bidirectionally to maintain on the one hand expression of *atonal1* in the rhombic lip and on the other hand, to specify the early expression of RP-derived choroid plexus markers such as *Transthyretin* [102]. In the hindbrain of zebrafish embryos, the interface between the squamous RP and the columnar rhombic lip epithelia is populated by a distinct *Gdf6a*-positive cell type, which was termed ‘veil cell’. Notably, veil cells contribute to RP expansion; they are able to generate squamous RP cells by direct transformation that occurs predominantly in the lower rhombic lip region and is accompanied by the downregulation of *Gdf6a*. Veil cells undergo both symmetric divisions that account for self-renewal and also asymmetric divisions that generate both types of progeny [105]. Hence, the majority of the hindbrain-level RP in several species derives from a *Gdf*-expressing lineage [102,105,106]. Although in the RP at spinal cord levels, *Gdf7* is also expressed in two lateral bands flanking a central domain [50,102], we still ignore whether these *Gdf7*-positive cells function as signaling boundaries to induce adjacent interneuron progenitors and/or act on the central RP, or whether they differentiate into derivatives distinct from those of the central domain.

## 4. Possible Mechanisms Leading to the Transition between Neural Crest and Roof Plate

Little is known about the timing of RP specification and differentiation vis-a-vis the period of NC production and emigration from the NT. Evidence suggests that BMP signaling is necessary for early development of both populations [12,93,95,107]. For example, overexpression of BMP or its downstream effector Msx1 showed that NC/RP cells expressing *BMP4, Wnt1* and *Lmx* can be induced up to stage 12HH, yet at later stages, dorsal progenitors lose their competence to generate these cell types and instead generate dorsal interneurons via a *Msx3*-dependent mechanism [98]. In favor of a restricted time window of responsiveness, we reported that, despite both NC and RP progenitors being initially dependent on BMP activity, the nascent RP becomes refractory to BMP (Figure 1B), likely a prerequisite for the end of NC emigration and the ensuing re-epithelialization of the dorsal NT that characterizes the RP stage [12]. Hence, dorsal neural progenitors, similar to ventral NT progenitors, exhibit a changing sensitivity to local morphogens over time [12,108].

In this context, we reported that BMP is necessary for initial expression of *Hes/Hairy* in the nascent RP, which in turn, downregulates responsiveness to BMP and reduces G1/S transition of premigratory NC, a prerequisite for cell emigration [12,30]. This is consistent with the observed constitutive mode of *Hes* gene expression in RP, which is associated with a lack of cell proliferation in this and other boundary cell types [109]. Along this line, Hes1 exhibits an oscillatory behavior in breast cancer cells and a relationship between *Hes1* dynamics and the cell-cycle was found such that in most cells, division takes place at or near the peak of *Hes1* expression. This peak in Hes1 protein expression is then followed by a dip, the onset of which is followed by the G1-S transition, leading to a second period of increase in Hes1 protein concentration before the next division. When *Hes1* oscillations were dampened, the cell-cycle slowed down, indicating the functional significance of *Hes1* oscillations for an efficient cell-cycle progression [110]. An intriguing question that awaits future investigation is whether premigratory NC cells express no *Hes* at all, or alternatively, whether they exhibit an oscillatory behavior of this gene, characteristic of cycling progenitors, which turns into a permanent mode of expression upon RP formation.

Important information on RP formation stems from the Dreher mutant mouse, a spontaneous neurological mutation defective in *Lmx1a* [111]. *Lmx1a* expression is restricted to the dorsal NT of wild-type mouse embryos encompassing both NC and RP phases of development (E8.5–E11.5). In Dreher mutants, while expression of *Lmx1a* begins normally and persists through E9.5, the NC stage, expression is not maintained through the mature RP. The loss of *Lmx1a* was accompanied by a complete failure of *Bmp6* and *Gdf7* expression throughout NT development, yet *Msx1* disappeared only by E11.5 and *Wnt1* and *Wnt3a* were not altered [112], suggesting that some features of the RP were maintained in the mutants. These results indicate that either *Lmx1a* is not necessary for all aspects of RP development or that additional factors or sources of Lmx-independent BMPs compensate for the loss of *Lmx1a*. In addition, in the hindbrain, only certain rostro-caudal regions of the RP are lost in the absence of *Lmx1a* [111,113], hinting at possible molecular heterogeneity in the responsiveness and properties of the hindbrain RP. This might also partially explain why the early differentiation of NC was normal at all axial levels of the developing spinal cord of Dreher embryos indicating that, despite its expression in NC, *Lmx1a* is not critically involved in the early NC program.

Another substantial signaling system is Notch-Delta, found to mediate the maintenance of the hindbrain RP epithelium [102]. This left open the question of possible role/s for this pathway in de novo RP formation. By gain and loss of Notch function in the trunk of quail and mouse embryos, respectively, we showed that initial formation of the RP crucially depends on Notch signaling, likely emanating from the RP-interneuron interface. Moreover, Notch signaling was found to be sufficient for the choice between RP and dI1 interneuron fates and necessary for the formation of the RP and dI1 interneurons (see Section 5.1). In contrast, it had no effect on the early development of NC [50]. Together, despite being highly significant for RP formation, neither Notch nor Lmx signaling affect the transition between NC and RP phases of dorsal NT development, leaving this transition open to investigation. This would favor the notion that despite being sequentially produced, separate signals are needed for ending NC production and for stimulating the emergence of a full repertoire of RP properties.

## 5. Fate and Functions of the RP

### 5.1. Fate

As previously shown, fate mapping analysis revealed that the lateral part of the RP generates the choroid plexus at the hindbrain level of the axis [101,102] (Figure 3). The transcription factor *Otx2* was found to be a master regulator of choroid plexus development and maintenance. Whereas conditional deletion of *Otx2* under the regulation of *Gdf7* affected primarily the hindbrain choroid plexus, deletion of *Otx2* by the *Otx2-Cre-ERT2* driver resulted in lack of all the plexi in the brain [114]. In addition, the RP is transformed into radial glia-like cells [115,116,117,118], that support growth of spinal cord axons [119]. These radial glial cells generate the stem cell-containing dorsal ependymal zone in the adult spinal cord of humans and rodents [115,120,121] (Figure 3). Recently, RP-derived canonical Wnt signaling was shown to promote ependymal cell proliferation in the dorsal midline of the spinal cord [115,121]. Notably, RNA profiling of the human and mouse spinal cords revealed that the mature ependymal zone maintains an embryonic-like dorsal pattern of expression of early NT transcription factors, such as *Msx1* and *Id4* and of signaling factors such as *BMP6* and *Gdf10*. A similar regionalization was reported for the ventral part of the ependymal zone (e.g, expression of *ARX, FoxA2*) [120].

In zebrafish, the dorso-ventral stretching of RP cells on their way to generate a radial glial scaffold, was shown to associate with the conversion of the primitive lumen into a central canal. Stretching of the RP was evidenced along the whole spinal cord with RP cells extending over 2/3 of the NT diameter. This process involves extension of the RP cytoskeleton and depends on activity of *Zic6* and Rho-associated kinase. Interestingly, *Mib* mutants defective in Notch signaling showed a loss of rostral RP cells [see also ref. [50] for mouse phenotype], absence of RP stretching and of a GFAP-positive radial scaffold [122]. In addition, several changes were documented during formation of the RP-derived ependymal layer in the spinal cord of mouse embryos; among them, a ventral expansion of BMP signaling and of *BMP receptor type 1B* expression was evident around the central canal lining with an associated reduction in Sonic hedgehog signaling [118]. Hence, fate of RP cells to become radial glia and the accompanying formation of the definitive central canal of the spinal cord are a source of significant morphogenetic changes in the NT and particularly in its dorsal domain.

In this context, it is worth elaborating on a poorly studied structure, termed the glycogen body, that is thought to derive from RP cells and is present across avian species (Figure 3). The glycogen body is an ovoid gelatinous mass characterized by the presence of glycogen filled cells [123,124,125]. It becomes first apparent around E7 on each side of the dorsal ependymal septum and fuses progressively into a single structure. In the chick, the glycogen body was originally defined as a structure restricted to the level of spinal nerves 25–29 (the “classical glycogen body”). Based on periodic acid-Schiff staining, glycogen body properties were reported to further extend all the way from cervical to coccygeal levels of the axis [126]. Nevertheless, the typical morphology of this structure remains confined to the lumbar region at all stages. The functions of the glycogen body remained elusive for many years. A recent study showed that by E10, no axons crossed the midline through this structure, albeit axonal decussation was apparent through the floor plate [127]. At the crural level, which is devoid of glycogen body, dorsal midline crossing was apparent through the RP, as it was at the cervical, brachial, thoracic and sacral levels. Such an accurate correlation suggests that the glycogen body serves as a physical barrier for axonal decussation at the sciatic plexus level. This differential axial behavior could account for hindlimb alternation whereas dorsal midline crossing at brachial levels correlates with synchronous wing movements. Thus, the glycogen body was postulated to be a major contributor to the alternating gait in birds, analogous to the molecular barriers to midline crossing reported in mice [127].

### 5.2. Functions

BMP family members have been found to mediate major functions exerted by the RP [128]. Manipulation of the level of BMP signaling in the NT showed that BMPs provide patterning information to both dorsal and intermediate cells. Within the resulting populations, graded BMP activities set expression domain boundaries of homeobox and basic helix-loop-helix (bHLH) families, ultimately leading to the generation of a diversity of differentiated neural cell types [11,94,107]. Furthermore, expression of *Math1* in vitro was induced by and subsequently became dependent upon BMP signaling [129,130].

To note is that signaling by BMPs is highly complex and time dependent. BMP7 was shown to be active at a post-patterning phase, when neural progenitors exit the cell cycle and enter the terminal differentiation pathway. In both avians and mice, BMP7 is required for the generation of dI1-dI3-dI5 interneurons, an activity mediated by Smad1 and Smad5, that operate through the canonical Smad pathway [131].

With regard to the Smad family of transcriptional BMP effectors, the presence and activity of inhibitory Smads, Smad6 and Smad7, was shown to be a possible contributing factor to limit the dorso-ventral extent of BMP action. Both Smad 6 and 7 are expressed in the NT and restrict the action of BMP signaling to its dorsal domain. For instance, ectopic misexpression of Smad7 suppressed dI1 and dI3 neural fates and concomitantly increased the number of dI4–dI6 spinal neurons. In contrast, Smad6 mostly blocked dI1 axon outgrowth. Taken together, these experiments suggest that inhibitory Smads have distinct roles in spatially limiting the response of cells to BMP signaling [132].

Wnt factors are also produced in the dorsal NT at both NC and RP stages. Absence of both *Wnt1* and *Wnt3a* caused a reduced number of dI1 and dI2 interneurons and a compensatory increase in dI3. This was mimicked by treatment of isolated neural plates with Wnt3a in a BMP-independent manner [97], highlighting the significance of Wnt signaling in interneuron development but not their timing of activity.

A more direct and time-controlled way to address the functions of the RP as a structure was its ablation by targeting diphtheria toxin under the regulation of *Gdf7*, an RP-specific gene. RP ablation had no effect on initial patterning of the dorsal NT resulting in normal NC development. Selective loss of the RP prevented the formation of both dI1 (*Math1*) and dI2 (*Ngn1*) interneurons and the dorsal midline was occupied instead by dI3 cells expressing *Mash1* [133]. suggesting an important function for the RP in development of dI1/2 interneuron populations.

In *Lmx1a* mutants (Dreher mice), however, *Math1*+ dI1 interneurons were generated in reduced numbers and no effect on dI2 was monitored [112]. At the level of the hindbrain, only the dorsal-most group of *Math1*-expressing neural progenitor cells, which comprise the rhombic lip, were lost [134]. This difference could be accounted for by the documented lack of *Gdf7*, *BMP6*, *Msx1* and *Wnt1* in the *Gdf7* mutants compared with the Dreher mice in which residual expression of *Wnt1* and *Msx1/2*, factors shown to operate on interneuron development, persisted.

In contrast to the spinal cord, less is known about functions of the RP at telencephalic levels. Using the Gdf7-diphteria toxin-mediated ablation paradigm, RP ablation resulted in a failure of midline induction and holoprocencephaly in the dorsal telencephalon. This was accompanied by a reduced activity gradient of BMPs. In dissociated cells and mutant explants, exogenous Bmp4 was sufficient to rescue RP-dependent midline patterning. Hence, the telencephalic RP is required for normal dorsal cortical patterning at least partially through BMP signaling [135].

Loss of Notch signaling in the dorsal NT, achieved by mutating the ubiquitin ligase *Mib* under the regulation of *Wnt-Cre*, also caused a specific loss of the RP with no apparent effect on the NC. In its absence, dI1 interneurons did not develop but dI2 were expanded up to the dorsal midline [50]. Differences in severity of dorsal interneuron phenotypes observed between the above experimental paradigms might suggest that early specification of interneuron subsets already begins prior to the advent of the definitive RP and is mediated by dorsal NT-derived BMPs or Wnts [94,131,136], or at later stages by BMPs derived from the ectoderm dorsal to the spinal cord [137]. In this context, it would be interesting to examine possible interactions between Notch signaling and members of the BMP and Wnt families at the various stages.

In addition to its role in dorsal interneuron development, the RP was shown to act as a barrier to axon growth. An early study reported that keratan sulfate is specifically expressed in the rat definitive RP and hypothesized that this and other glycosaminoglycans might inhibit bilateral crossing of incoming sensory or of commissural nerve fibers [138]. BMP7 and Gdf7 were later found to orient the initial ventral extension of commissural axons both in vitro and in mutant mice, and to carry out this repellent activity in the form of GDF7:BMP7 heterodimers [139]. As described in Section 5.1, this barrier-like activity may also be species-dependent, as at a later embryonic stage in birds, the RP-derived glycogen body may serve as a physical barrier for axonal crossing at the lumbar level of the neuraxis. This finding might bear evolutionary significance, as paleontological findings indicate that the glycogen body was already present in the lumbar spinal cord of dinosaur ancestors to all flying diapsids, such as pterosaurs and birds [140]. This would reinforce the notion of an ancestral co-evolution of a lumbar glycogen body that enables alternative movements of the hindlimbs vs. synchronous wing (forelimb) flapping [127].

## 6. Conclusions and Future Perspectives

During neural development, NC cells that generate the peripheral nervous system, and definitive RP cells of the central nervous system, are sequentially formed in the same anlagen. Thus, it is the dorsal domain of the NT where the major decision of becoming peripheral or central nervous system takes place. How the dorsal neural primordium transits between these phases remains largely unknown. Evidence suggests that these peripheral and central neural branches segregate from a common progenitor in the dorsal NT already prior to the completion of NC emigration [17,19]. Transcriptome analysis uncovered genes differentially expressed in NC and RP [50] and in RP and interneurons [141]; these will provide the basis for investigating the molecular networks responsible for fate transitions in the dorsal NT in association with the cellular and morphogenetic processes that this domain undergoes during maturation. Together with current knowledge of the main signaling systems involved in NC and RP formation, e.g, BMP, Wnt, RA and Notch factors, it will be possible to further pinpoint differential effects of factors driving the end of NC production and the beginning of definitive RP ontogeny. Much is still to be done to clarify the extent to which initial specification vs. subsequent differentiation of various interneuron cell types depends on changing properties of the dorsal NT between NC and RP stages.

Our knowledge of the fate and functions of the RP is growing. Along this line, investigating the heterogeneity of RP properties at various levels of the neuraxis and its functional significance are also a subject of considerable interest. The development of the dorsal NT embodies basic processes in development: the regulation of cell proliferation, cellular movements and cell elongation, epithelial-mesenchymal transitions, lineage decisions, and relationships between them. Aberrant signaling during critical phases leads to disease, ranging from defects in NT closure [142,143,144], lack of dorsal cell types [50,92,133], neurocristopathies [145,146], and tumors generated from RP derivatives such as choroid plexus papillomas or carcinomas [147]. Our expanding knowledge of basic mechanisms of development should assist us in developing animal models for addressing their etiology, prevention and treatment. In vivo approaches will undoubtedly be complemented with more accessible models that consist of embryonic stem (ES) cells or adult pluripotent stem cells induced to produce spinal organoids, elongated trunk-like structures composed of both neural and mesodermal derivatives, and assembloids containing defined tissues, all aimed at mimicking dorsal neural development in a dish [107,148,149,150].

## Data Availability

Not applicable.
